# Effects of Exercise Training on Cardiac Mitochondrial Functions in Diabetic Heart: A Systematic Review

**DOI:** 10.3390/ijms26010008

**Published:** 2024-12-24

**Authors:** Iqbal Ali Shah, Shahid Ishaq, Shin-Da Lee, Bor-Tsang Wu

**Affiliations:** 1 PhD Program in Healthcare Science, China Medical University, Taichung 40402, Taiwan; u112313102@cmu.edu.tw (I.A.S.); shahidishaq53@gmail.com (S.I.); 2 Department of Physical Therapy, China Medical University, Taichung 40402, Taiwan; 3 Department of Senior Citizen Service Management, National Taichung University of Science and Technology, Taichung 40343, Taiwan; wusletter@nutc.edu.tw

**Keywords:** diabetic heart, exercise training, mitochondrial biogenesis, mitochondrial dynamics, mitochondrial function, mitochondrial oxidative phosphorylation

## Abstract

A diabetic heart is characterized by fibrosis, autophagy, oxidative stress, and altered mitochondrial functions. For this review, three databases (PubMed, EMBASE, and Web of Science) were searched for articles written in English from September 2023 to April 2024. Studies that used exercise training for at least 3 weeks and which reported positive, negative, or no effects were included. The CAMARADES checklist was used to assess the quality of the included studies, and ten studies (CAMARADES scores 4–7/10) were included. Nine studies showed that exercise training improved cardiac mitochondrial oxidative phosphorylation by decreasing ROS, increasing electron transport chain activity, and enhancing the production of ATP. Eight studies indicated that exercise training ameliorated mitochondrial biogenesis by increasing the levels of AMPK, PGC-1α, Akt, Irisin, and Sirtuin-III. Moreover, four studies focused on mitochondrial dynamics and concluded that exercise training helped decrease the levels of mitochondrial fission factor and dynamin-related protein- 1. Finally, six studies revealed improvements in mitochondrial physiological characteristics such as size, potential, and permeability. Our findings demonstrate the beneficial effects of exercise training on cardiac mitochondrial function in diabetic hearts. Exercise training improves cardiac mitochondrial physiological characteristics, oxidative phosphorylation, biogenesis, and dynamics.

## 1. Introduction

People with diabetes have impaired insulin sensitivity. In the heart, sensitivity to insulin becomes dysfunctional at different levels; for example, decreased insulin-activated glucose uptake with decreased glucose transporter (GLUT-4) expression results in the reduced transport of GLUT-4 from the cytoplasm to the cell membrane, leading to a diabetic heart [1]. The hallmarks of a diabetic heart include fibrosis, impaired autophagy, apoptosis, oxidative stress, inflammation, and altered calcium handling [2]. Diabetic cardiomyopathy (DCM) affects the heart both functionally and structurally [3]. In a diabetic heart, the physiological properties of mitochondria, such as size, shape, and membrane potential are affected, as is the ratio between the cristae and mitochondrial areas [4]. In the hearts of mice with diabetes mellitus, exercise training for 6 weeks has been shown to improve overall sarcomere structures and increase mitochondrial cross-sectional area, circumference, and Feret’s diameters, while promoting the characteristics of the inner mitochondrial membrane and mitochondrial morphology [5]. Mitochondria are important cell organelles that not only play a role in energy production but also contribute to different biological processes such as growth and redox reactions [6]. The diabetic heart exhibits an altered mitochondrial morphology, as well as decreases in size, complexity, and membrane potential [7]. Furthermore, mitochondrial changes in the diabetic heart decrease the production of adenosine triphosphate (ATP) due to reduced activity of the electron transport chain (ETC), lower respiratory control ratio (RCR) values, and lower levels of superoxide di mutase (SDo2) [8]. The production of reactive oxygen species (ROS) in excess amounts leads to oxidative stress in cells, further decreasing oxidative phosphorylation [9]. Exercise training has been shown to increase oxidative phosphorylation in diabetic rats by making glucose a substrate readily available to these animals. Therefore, exercise aids in preventing certain cardiometabolic abnormalities linked to diabetes [10].

Mitochondrial biogenesis is activated and regulated by peroxisome proliferator-activated receptor gamma co-activator-1 alpha (PGC-1α), which binds to and triggers different transcription factors such as PPARα, PPAR-γ, and NRF (1 and 2). NRF2 activates mitochondrial transcription factor A (TFAM), which further drives the transcription and reproduction of mitochondrial DNA (mt DNA), thereby increasing biogenesis [11]. Altering the ratio of adenosine monophosphate to adenosine triphosphate (AMP/ATP) activates the PGC-1α-Nrf1/2-TFAM pathway. An increased level of AMP leads to a rise in AMPK concentration, which causes PGC-1α phosphorylation, thus increasing gene expression in mitochondria and accelerating mitochondrial biogenesis [12]. Mitochondrial biogenesis has been shown to be significantly reduced in diabetic mice, as evidenced by reduced transcription of PGC-1α, NRF, and TFAM genes [13]. Researchers have also found that genes encoding for mitochondrial biogenesis, such as PGC-1α and β, are downregulated in diabetic hearts [14]. In diabetic rats, exercise training has been shown to restore mitochondrial biogenesis and the replication of mitochondrial DNA to a certain degree by regulating PGC-1α and its associated transcription factors [15].

Mitochondrial dynamics consist of fission and fusion. Fusion is controlled by proteins such as optic atrophy 1 (opa1) and Mitofusion 1 and 2 (Mf1 and 2), while fission is regulated by dynamin-related protein (Drp1), mitochondrial fission factor (MFF), and mitochondrial fission protein 1 (Fis1) [16]. The authors of [17] found that Opa1 knockout mice exhibited cardiomyopathy with distinct presentations, such as mitochondrial dysfunction and decreased cardiac function, while the deletion of Mf1 and Mf2 caused excessive mitochondrial breakdown and life-threatening cardiomyopathy. Mitochondrial dynamics are altered in the diabetic heart. Hyperglycemia causes H9c2 cardiac myoblast cells to produce short, small mitochondria that primarily rely on Drp1 phosphorylation [17]. The dominant negative Drp1 mutant inhibits mitochondrial fission, while cell mortality is worsened by high glucose-induced ROS generation [18]. Additionally, in one study, coronary cells taken from the hearts of mice with streptozotocin-induced diabetes were found to exhibit elevated mitochondrial fission, decreased Opa1, and increased Drp1 [19]. However, exercise training has been shown to improve cardiac mitochondrial dynamics by regulating fission and fusion, which in turn improves cardiac mitochondrial function [20].

Exercise training improves the diabetic heart by increasing levels of adenosine triphosphate (ATP), PGC-1α, TFAM, membrane potential, and superoxide dismutase, while decreasing levels of reactive oxygen species (ROS) and uncoupling proteins (UCP II and III) [21]. However, there exists a controversy, in that some studies have shown that TFAM levels increase in response to exercise training, while others have shown no changes in the levels of TFAM [22]. The intricate molecular mechanisms of cardiac mitochondrial function in diabetic hearts and the impact of exercise on this function make it a pressing subject in need of further investigations. Therefore, this systematic review was conducted to determine the effects of exercise on cardiac mitochondrial function in a diabetic heart, specifically focusing on mitochondrial oxidative phosphorylation, biogenesis, dynamics, physiological function, glycemic parameters, and body weight.

## 2. Materials and Methods

### 2.1. Protocol and Registration

This systematic review was registered in the research registry with ID no. (reviewregistry1788). In addition, the Preferred Reporting Items for Systematic reviews and Meta-Analyses (PRISMA) checklist was followed in order to maintain the quality of the review.

### 2.2. Information Sources

Related studies were searched for related articles from three databases: PubMed, Web of Science, and EMBASE. Further articles were obtained from the reference lists in the obtained articles. This search was carried out from September 2023 to April 2024.

### 2.3. Study Design

Control animal trials written in English were included in this systematic review. No restrictions were applied concerning publication date. Studies with a treatment duration of less than three weeks or lacking relevant outcomes such as mitochondrial oxidative phosphorylation, physiological characteristics, biogenesis, and dynamics, were not included. In addition, studies involving interventions other than exercise, such as pharmacological or dietary treatments, or those combining exercise with other interventions, were also excluded.

### 2.4. Animal Model Type

Studies in which animals, such as rats and mice, were induced with diabetes and exhibited diabetic heart or diabetic cardiomyopathy were included in this review, with specifications concerning age, type of species, and gender.

### 2.5. Intervention Focus

Studies involving exercise of any type, regardless of frequency or duration, were included; such exercise included aerobics, high-intensity and low-intensity interval training, and short-term and resistance exercise (performed either through running on a treadmill, climbing a ladder, or running on a motorized exercise wheel system) for at least three weeks.

### 2.6. Comparators

Studies with at least a normal (sham or not), negative (sedentary diabetic heart), and positive (exercise-trained diabetic heart) group were included.

### 2.7. Outcomes

To assess the effects of exercise training on cardiac mitochondrial function in diabetic hearts, the primary outcomes measured were mitochondrial oxidative phosphorylation and mitochondrial biogenesis. These outcomes were assessed based on changes that occurred in the levels of certain proteins and enzymes; these included electron transport chain complex (CI, CII, CIII, CIV, and CV complex) enzymes, mRNA, collagen types I and III, NADH, PGC-1α, AMPK, Irisin, and TFAM, as well as other proteins and enzymes involved in these reactions.

The secondary outcomes assessed were mitochondrial physiological properties, dynamics, glycemic parameters, and body weight. The dynamics included fission and fusion. The physiological characteristics included the shape, size, density, permeability, and potential of mitochondria. Glycemic parameters indicated the level of glucose in blood. Body weight indicated body size and structure.

### 2.8. Search Strategy

The search strategy involved keywords composed of the following three areas of specification: (1) exercise training OR physical activity; (2) mitochondrial function OR mitochondrial oxidative phosphorylation OR mitochondrial biogenesis OR mitochondrial dynamics OR mitochondrial physiological properties; and (3) diabetic heart OR diabetic cardiomyopathy. Using Boolean operators, the terms were combined as follows: (exercise training OR physical activity) AND (mitochondrial function OR mitochondrial oxidative phosphorylation OR mitochondrial biogenesis OR mitochondrial dynamics OR mitochondrial physiological properties) AND (diabetic heart OR diabetic cardiomyopathy).

### 2.9. Selection Process

Two independent appraisers screened the abstracts and titles of the initially identified studies for duplication and relevant topics. Subsequently, the same appraisers thoroughly reviewed the full texts based on the above-mentioned inclusion criteria. Decisions were finalized after discussion with a third evaluator to address any disagreements.

### 2.10. Data Management

All the studies of this systematic review were kept in an Excel file. Tables, graphs, and write-ups were also saved, in separate files, so that they could be retrieved when needed for further clarification and review processes.

### 2.11. Data Collection Process

All the data obtained in this systematic review were collected by thoroughly reading the texts, graphs, and charts in the methods and results sections of the included studies.

### 2.12. Data Items

According to the study characteristics, we included the last name of the first author, the animal model used (type of species and age), and the exercise used (frequency and duration). For the outcomes, we included the results for the cardiac mitochondrial functions, specifically the oxidative phosphorylation, biogenesis, dynamics, and physiological characteristics. We also included the results for glucose level and body weight. Two independent evaluators discussed and analyzed the results, and no assumptions were made regarding missing and confusing data.

### 2.13. Outcomes and Prioritization

The two primary outcomes of this systematic review were mitochondrial oxidative phosphorylation and mitochondrial biogenesis. These were selected as they involve the processes of converting food to energy, which helps cardiomyocytes to synthesize and generate ATP and maintain their morphology. Four more outcomes were also assessed; these were mitochondrial physiological properties, mitochondrial dynamics, glycemic control, and body weight.

### 2.14. Risk of Bias Assessment

To maintain the quality of this systematic review, two researchers independently assessed the included studies, using a collaborative approach to meta-analysis and a review of animal data from the checklist of experimental studies (CAMARADES), which is a 10-item checklist that assesses the risk of bias in pre-clinical animal studies. The total score for this review was found to be in the range of 4–7/10. The CAMARADES checklist is presented below, in Table 1.

### 2.15. Data Synthesis

**Figure 1:**
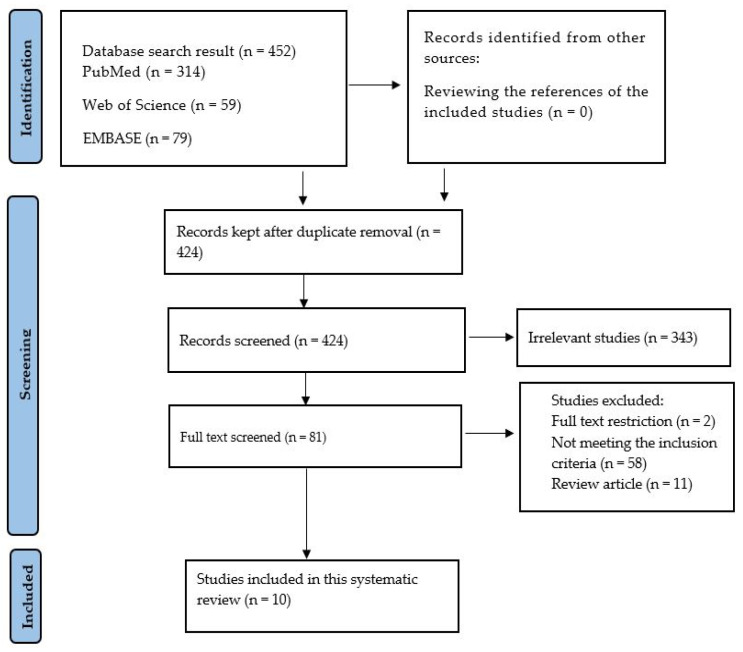
A PRISMA chart indicating the procedure for the inclusion of studies.

The process of data collection was illustrated using a PRISMA flow chart diagram (Figure 1). The study characteristics included the name of the first author, the animal model, the exercise type, and the type of diabetes induced. The effects of exercise on mitochondrial function in diabetic hearts were indicated in texts, tables, and graphs using signs for increase (↑), decrease (↓), and no change (NC), in measurement variables and in the frequencies with which outcomes were presented (the number of outcomes in which an increase or decrease was recorded).

## 3. Results

### 3.1. Search Results

At the very beginning, the search contained 452 articles from three different databases: PubMed (n = 314), Web of Science (n = 59), and EMBASE (n = 79). After duplicate removal (n = 28), a total of 424 studies were screened. The screening focused on abstracts and titles. As a result, 343 articles were found to be unrelated; these were then removed. The remaining 81 articles were then fully read. Of these eighty-one studies, fifty-eight did not meet the inclusion criteria; in addition, two were removed for being abstracts, and eleven were removed for being review articles. As a result, only 10 studies were left to be included in this systematic review.

### 3.2. Effects of Exercise Training on Cardiac Physiological Properties in Diabetic Hearts

Cardiac physiological characteristics (Table 2) mainly focused on size, shape, area, density, membrane permeability, and mitochondrial membrane potential. Two of the studies focused on mitochondrial size and area [5,26]; these found that the size and area of mitochondria were both reduced in diabetic hearts but were increased by exercise training. Both of these studies [5,26] demonstrated that density was not significantly affected in diabetic models and was not significantly influenced by exercise. Mitochondrial membrane potential (MMP) was discussed in two studies [21,24], both of which showed that MMP was decreased in diabetic hearts but increased with exercise training. In two further studies, membrane permeability was found to be abnormal [27] and decreased in diabetes [29], but restored and increased with exercise training.

### 3.3. Effects of Exercise Training on Cardiac Oxidative Phosphorylation in Diabetic Hearts

Three studies [5,21,24] demonstrated that reactive oxygen species (ROS) production was increased in diabetic hearts but reduced with exercise training. The respiratory chain reaction (RCR) was reported to decrease in diabetic hearts in two studies [21,24], but was found to increase in another work [29]. In the former two studies, exercise training was shown to increase RCR; in the latter, a decrease in RCR after exercise training was reported. Four studies focused on ATP production [5,24,27,28]; all reported that ATP production was decreased in diabetic hearts but enhanced by exercise training. Electron transport chain (ETC) complex activity was investigated in five studies [5,22,24,26,29]. Four of these reported decreased activity in ETC enzymes in all components, while one study [29] showed decreased activity in complexes I and II only. Exercise training was found to increase ETC activity in all components; however, one study found that exercise training resulted in decreased activity in two components, increased activity in one component, and no change in the other two components. SOD2 was reported to be decreased in diabetic hearts in four studies [5,21,22,24], three of which reported that SOD2 was increased with exercise training, with one [22], reporting a decreased level of SOD2. Production of UCP2 [21,24], was also found to be increased in diabetic hearts but reduced by exercise [5,22]. The oxygen consumption rate (OCR) was found to decrease in one study [24] but to increase in another work [27]. In both these studies, a diabetic model was used; however, exercise training was reported to increase OCR in the former paper, but to decrease OCR in the latter. Respiratory control ratio (RCR) was targeted by three [21,24,29] studies, all of which showed a decreased level of RCR in diabetic hearts but an increased level after exercise training.

### 3.4. Effects of Exercise Training on Cardiac Mitochondrial Biogenesis

Cardiac mitochondrial biogenesis (Table 2) was targeted in eight studies. Three of these studies [5,23,24] indicated that the level of AMP-activated protein kinase (AMPK) was reduced in diabetic hearts but increased with exercise training. In addition, four studies [21,22,24,25] elaborated the effects of exercise on peroxisome proliferator-activated receptor-gamma-1 alpha (PGC-1α), and all showed that exercise training increased the level of PGC-1α. The level of mitochondrial DNA (mt DNA) [22,25], was found to be reduced in diabetic hearts, but increased by exercise training. Diabetic hearts exhibited decreased levels of mRNA for genes of biogenesis such as SSDBP1, Twinkle, and Top1mt, while their levels increased with exercise [25]. The mitochondrial transcription factor (TFAM) was found to be at low levels in diabetic hearts in four studies [21,22,25,29]. Exercise training was found to increase TFAM levels in three of these studies [21,25,29]; in the other work, [22], no change in TFAM concentration was recorded. Decreased concentrations of AKT [25], Sirtuin-3 [5], and Irisin [23] were also reported in diabetic hearts, with increased levels resulting from exercise. Finally, two studies focused on nuclear respiratory factor (NRF 1 and 2) [22,25], and obtained contradictory results: one study reported increases in levels of NRF 1 and 2 [25], while the other showed no changes [22].

### 3.5. Effects of Exercise Training on Cardiac Mitochondrial Dynamics in Diabetic Hearts

Mitochondrial dynamics (Table 2) characterized by fission and fusion were assessed in three studies [22,23,27]. Dynamin-related protein 1(Drp1) was assessed in two studies [23,27]; these reported that the Drp1 level was increased in diabetic hearts but lowered by exercise training. One study [23] focusing on mitochondrial fission factor (MFF) and Fis1 found that these factors were increased in diabetic hearts, but reduced with exercise training. In another study [22], signs of fission and fusion were found in diabetic hearts, but their levels were not significantly affected by exercise.

### 3.6. Effects of Exercise Training on Glycemic Parameters and Body Weight

Glycemic parameters and body weight were discussed in all of the included studies, as shown in Table 2. Contrasting results were observed, as five of the studies [5,23,27,28,29] reported that diabetic hearts had elevated levels of glucose and increased body weight, and that exercise training caused significant reductions in body weight and levels of plasma glucose. However, one study [26] found only slight decreases in body weight and glucose levels. Another study [26] focused on body weight and reported a significant reduction in exercise models compared with diabetic hearts. One other study [21] confirmed that body weight remained unchanged while glucose levels increased in diabetic hearts as compared to exercise group. Moreover, another study [25] demonstrated that, in diabetic hearts, exercise did not result in any change in either body weight or glucose levels. Finally, the authors of [23], found that glucose levels and body weight decreased [29] in hyperglycemic hearts, but their levels increased with exercise training.

## 4. Discussion

**Figure 2:**
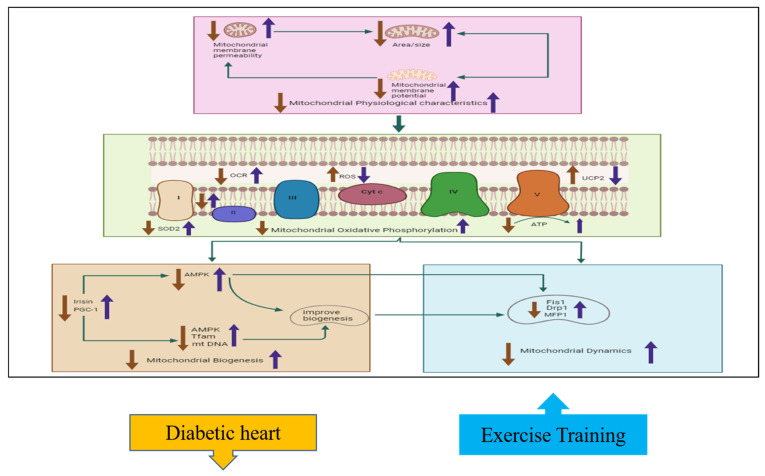
The effects of exercise on cardiac mitochondrial function, including physiological characteristics, oxidative phosphorylation, biogenesis, and dynamics. This hypothesized figure shows how exercise training ameliorates mitochondrial functions by affecting different parameters. The physiological properties, including mitochondrial membrane potential, permeability, and mitochondrial size, decreased in diabetic hearts but increased with exercise training. Mitochondrial oxidative phosphorylation characteristics are also improved with exercise training; such improvements include increased SOD2, ATP, and complex activity, with decreased UCP2 and ROS levels. Mitochondrial biogenesis factors such as PGC1 and irisin activate AMPK, Tfam, and mt-DNA; these in turn improve biogenesis by reducing the expression of the mitochondrial Drp1, MFP1, and Fis1 factors. The blue arrow indicates increase with exercise training while the orange color indicates decrease in diabetic heart of the different parameters involved in mitochondrial function.

Our findings demonstrate the beneficial effects of exercise training on cardiac mitochondrial function in diabetic hearts as indicated in Figure 2. The physiological characteristics of diabetic hearts improve with increases in size and membrane potential and the restoration of membrane permeability. Cardiac mitochondrial oxidative phosphorylation is enhanced in diabetic hearts with increased ETC enzymatic activity and ATP production and decreased OCR and ROS production. Cardiac mitochondrial biogenesis is upregulated in diabetic hearts by increased levels of AMPK, PGC-1α, TFAM, Twinkle, top1mt, SSDBP1, Irisin, mt DNA, and Sirtuin-III. Cardiac mitochondrial dynamics are improved in diabetic hearts by decreased levels of Drp1, Fis1, and MFF. Collectively, these findings reveal that exercise training has beneficial effects on cardiac mitochondrial function in diabetic hearts.

Cardiovascular diseases are associated with structural changes in the mitochondria of cardiac tissue, including the formation of large-size mitochondria ranging up to 30 μm [30], while exercise training improves mitochondrial size in diabetic hearts [24]. Studies [7,31] have revealed that cardiac mitochondrial size and area decrease in diabetic mice but increase with exercise training [5,26]. Cardiac mitochondrial membrane potential was decreased by fifty-four percent in diabetic mice [32], but increased by treadmill training and ladder climbing. In diabetic rats, the membrane permeability of cardiac mitochondria exhibited an increased sensitivity [33], which was restored and enhanced following exercise training [27]. Moreover, in one review article [34], it was shown that diminished mitochondrial density was observed in diabetic hearts, in contradiction to our own finding that density is not affected by diabetes and exercise training [26].

Diabetes-associated metabolic disorders lead to cardiovascular disorders, and the characteristic features includes mitochondrial dysfunction, impaired electron transport chain activity, and increased ROS production [35], while exercise training reduces mitochondrial dysfunction, increases electron transport chain activity, and decreases ROS production in diabetic hearts [23]. Review studies [36,37] have demonstrated decreased oxidative phosphorylation due to reduced ATP generation, ETC enzymatic activity, and increased ROS production in type-I and type-II diabetic hearts, while exercise training has been shown to increase ATP production and ETC enzyme activity and reduce ROS production [24]. In one study, superoxide dismutase (SOD2) was substantially reduced in diabetic mice [31], while three other studies [5,21,24] reported that exercise training increased its levels. However, one study [22] indicated that SOD2 was reduced as a result of exercise training [22]. Mitochondrial uncoupling proteins II and III ameliorated oxidative stress and acted as antioxidants in diabetic hearts [38]. However, resistance exercise and treadmill running reduced the expression of UCPII and III [21]. Another study focusing on the oxygen consumption rate of myocardial cells revealed that it was reduced in diabetic hearts, leading to a decrease in cardiac efficacy [39]. This finding contradicted our results, which indicate that exercise training could enhance oxygen consumption [27], while another study reported a reduction in oxygen consumption with exercise training [24].

Proteins involved in mitochondrial biogenesis such as PGC-1α, AKT, TFAM and Sirtuin are reduced in ischemic heart disease [40], while exercise training upregulates PGC-1α, AKT, and (TFAM) level in diabetic hearts [23]. Studies [41,42] have also demonstrated that hyperglycemia reduces the mRNA level of PGC-1α in cardiac H9C2 cells, and decreases irisin levels in diabetic hearts, whereas exercise training increases PGC-1α and irisin levels, in turn increasing mitochondrial biogenesis [23,24]. Two studies have reported that levels of AMPK, mtDNA, and TFAM are decreased in diabetic hearts [43,44], while other studies have reported that exercise training increases levels of PGC1α, irisin, and TFAM [23,24]. In another study, it was reported that Sirtuin 1 and 3 expression was reduced in rats fed on a high-fat diet, compared with control rats [45], while exercise training was reported to increase Sirtuin levels in another work [5]. Akt translocation to myocardial cells was found to be disturbed in both type I and II diabetic mice [46], whereas exercise training increased Akt level and its translocation to myocardial cells. The expression of mitochondrial transcription genes such as Twinkle, top1mt, and SSDBP1 were reduced in hypertrophy and heart failure due to diabetes, which further caused reduced mitochondrial biogenesis [47], while exercise training increased the expression level of mitochondrial transcription genes [25].

Changes in mitochondrial dynamics proteins involved in fusion and fission, such as Mitofusion1 and 2 (MFN1 and 2), OPA 1, DRP1, and FIS1, are associated with diabetes and cardiovascular disorders [48], while exercise training upregulates fusion factors and downregulates fission factors in diabetic hearts [25,29]. The authors of [46] found that, in the hearts of diabetic mice, levels of the Mitofusion 2 gene declined, while those of other genes such as Drp1, Mfn1, Fis1, and Opa1 remained unchanged, leading to excessive mitochondrial fission [49]. This finding contradicts our results, which showed increased Drp1, Fis1, and MFF levels in diabetic hearts, with exercise training lowering their levels [23]. Mitochondrial fusion was decreased and fission was increased in five-week diabetic mice [50], while exercise training showed no effect on fusion and fission [22].

In the future, for studies of diabetic humans undergoing exercise training, advanced imagining tools such as computerized tomography (CT) angiography, supra-aortic trunk ultrasound, nuclear imagining techniques, and magnetic resonance image (MRI) could be used to provide detailed assessments of myocardial structures and functions [51]. Future research should focus on clinical trials to see the effects of exercise training on cardiac mitochondrial functions in diabetic heart disease.

### Limitations

Some of the limitations of this systematic review have been identified. First, this systematic review includes articles written in English and sourced from three databases: PubMed, EMBASE, and Web of Science. Second, the coverage of mitochondrial aspects varies significantly, with mitochondrial oxidative phosphorylation discussed in nine studies, mitochondrial biogenesis in eight studies, mitochondrial dynamics in four studies, and mitochondrial physiological characteristics in six studies. Third, the review considers studies only with a treatment duration of more than three weeks, which limits our ability to assess the immediate effects of exercise training on the diabetic heart. Fourth, gender-based analysis was not conducted because the included studies exclusively involved male rats and mice.

## 5. Conclusions

The results of our systematic review clearly indicate that exercise training can improve diabetic hearts because (I) exercise training enhances cardiac mitochondrial oxidative phosphorylation; (II) exercise training improves cardiac mitochondrial biogenesis; and (III) exercise training restores mitochondrial physiological properties and ameliorates fission and fusion. Based on these points, we conclude that exercise training helps control the death of cardiac myocytes by improving cardiac mitochondrial function. Therefore, we can assert that exercise is a suitable and feasible method for protecting against diabetic heart conditions.

## Figures and Tables

**Table 1:** The CAMARADES Checklist.

References	1	2	3	4	5	6	7	8	9	10	Total
1. Wang et al. [23]	√	√	√			√	√		√	√	7
2. Wang et al. [24]	√					√	√		√	√	5
3. Wang et al. [25]	√		√			√	√		√	√	6
4. Bækkerud et al. [26]	√					√	√			√	4
5. Veeranki et al. [27]	√					√	√			√	4
6. Mokhtar et al. [28]	√	√	√			√	√				5
7. Jin et al. [5]	√	√	√			√	√		√	√	7
8. Ko et al. [21]	√	√				√	√		√		5
9. Botta et al. [22]	√	√	√			√	√		√		6
10. Oliveira et al. [29]	√	√	√			√	√		√		6

1. Publication in peer review journal. 2. Statement of control of temperature. 3. Randomization of treatment or control. 4. Allocation concealment. 5. Blinded assessment of outcome. 6. Avoidance of anesthetics with marked intrinsic properties. 7. Use of animals with diabetes or diabetic cardiomyopathy. 8. Sample size calculation. 9. Statement of compliance with regulatory requirements. 10. Statement regarding possible conflicts of interest. √—yes.

**Table 2:** Animal samples, diabetes types, exercise types and parameters, glycemic parameters, and mitochondrial characteristics in response to exercise.

References	Sample and Diabetes Type	Exercise Type	Exercise Parameters	Glycemic Parameters and Body Weight (BW)	Mitochondrial Physiological Characteristics	Mitochondrial Oxidative Phosphorylation	Mitochondrial Biogenesis	Mitochondrial Dynamics
1. Wang et al. [23]	**S:** Male Wistar rats **A:** 48 weeks **DT:** Type-II	Treadmill training	**Sp:** 10–18 m/min **D:** 10–45 min/day **F:** 5 days/week **P:** 8 weeks	**DH**: BW↑ ↑,hyperglycemia↑ **ET**: BW↓, hyperglycemia↓		**DH**: NADH↓, SDH↓, CCR↓, CCO↓ **ET**: NADH↑, SDH↑, CCR↑,CCO↑	**DH**: AMPK↓ Irisin↓ **ET**: AMPK↑, Irisin↑	**DH**: Drp1↑, Fis1↑,MFF↑ **ET**: Drp1↓, Fis1↓,MFF↓
2. Wang et al. [24]	**S:** Male C57BL/6 mice **A:** 6 weeks **DT:** Type-II	Treadmill training	**Sp:** 10 m/min **D:** 1 hr/day **F:** 4 days/week **P:** 16 weeks	**DH**: BW↑, plasma glucose↑ **ET**: BW↓, ↓plasma glucose	**DH**: Membrane potential↓ **ET**: Membrane potential↑	**DH**: 4HNE↑,Nox4↑,p47phox↑, p67phox↑,ROS↑ **ET**: 4HNE↓,Nox4↓, p47phox↓, p67phox↓,ROS↓ **DH**: UCP2↑, ATP↓, RCR↓, OCR↑ **ET**: UCP2↓, ATP↑, RCR↑, OCR↓ **DH**: ETC enzymes like NUDE↓, CCR↓, COX↓, SDH↓, SOD2↓ **ET**:ETC enzymes like NUDE↑, CCR↑, COX↑,SDH↑,SOD2↑	**DH**: PGC-1α↓,AMPK↓ **ET**:PGC-1α↑,AMPK↑	
3. Wang et al. [25]	**S:** Male C57BL/6 mice **A:** 6 weeks **DT:** Type-II	Treadmill training	**Sp:** 10 m/min **D:** 1 hr/day **F:** days/week **P:** 15 weeks	**DH**: BW↑ **ET**: BW↓			**DH**: 16sRNA↓,ND1↓,ND6↓, CYBT↓,mtDNA↓,SSDPB1↓,Top1mt↓ Twinkle↓ **ET**: 16sRNA↑,ND1↑,ND6↑, CYBT↑,mtDNA↑,SSDPB1↑, Top1mt ↑, Twinkle↑ **DH**: PGC1α↓,AKT↓,TFAM↓ **ET**: PGC1α ↑,AKT↑,TFAM↑ **DH**: mRNA of NRF1↓ **ET**: mRNA of NRF1 ↑	
4. Bækkerud et al. [26]	**S:** Male (BKS.cgm+/Lepdb/Bom Tac) db/db mice **A:** 8 weeks **DT:** Type-II	Treadmill training	**Sp:** progressive 0.03 m/s **D:** 40 min/day **F:** 5 days/week **P:** 8 weeks	**DH**:BW↑,serum glucose↑ **ET**: BW↓,No significant change In glucose level	**DH**: Size↓,quantity↑ and density (no change) **ET**: Slight ↑ in size and quantity and NC in density	**DH**: ETC complexes (CI, I+II, III, IV) activity↓ **ET**: ETC complexes (CI+II,CII and CIV) Activity↑ **DH**: IDH↑,OGDH↑,SDH↑, **ET**: IDH↓,OGDH↓,SDH↓ **DH**: MCU↓ **ET**: MCU ↑		**DH**: MFP1↓ **ET**: MFP1↑
5. Veeranki et al. [27]	**S:** Male C57BL/6 mice **A:** 2 months **DT:** Type-II	Treadmill training	**Sp:** 10–11 m/min **F:** 5 day/week **P:** 5 weeks	**DH**: BW↑, glucose↑ **ET**: BW↓, glucose↓	**DH**: Abnormal membrane permeability **ET**: Restores membrane permeability	**DH**: OCR↓, ATP↓ **ET**: OCR↑, ATP↓ **DH**: Cytochrome content↓, leakage↑ **ET**: Cytochrome content↑, leakage↓	**DH**: Mitochondrial biogenesis imbalance **ET**: Mitochondrial biogenesis balanced	**DH**: Drp1↑,Mfn2(NC) **ET**: Drp1,Mfn2 (NC)
6. Mokhtar et al. [28]	**S:** Male Wistar rats **A:** N/A **DT:** Type-II	Rodent treadmill training	**Sp:** Progressive exercise **D:** 10–60 min/day **F:** 5 days/week **P:** 10 week	**DH**: Same BW, glucose↑ **ET**: No changes in BW and glucose levels		**DH**: State-3 respiration↓, ATP ↓ **ET**: State-3 respiration↑, ATP↑		
7. Jin et al. [5]	**S:** Male C57BL/6J mice **A:** 6 weeks **DT:** Type-II	Treadmill training	**P:** 6 weeks	**DH**: BW↑, fasting blood glucose↑ **ET**: BW↓, fasting blood glucose↓	**DH**: Area↓, diameter↓, perimeter↓, cristae area↓, mitochondrial area↓ **ET**: Area↑, diameter↑, perimeter↑, cristae area↑, mitochondrial area↑	**DH**: oxidative stress↑, ATP↓, FAO↓, ROS↑ **ET**: oxidative stress↓, ATP↑, FAO↑, ROS↓ **DH**: MRC complex (I -V) activity↓ **ET**: MRC complex(I-V) activity ↑ **DH**: Mitochondrial acetylation of SOD2↑, LCAD ↑ **ET**: Mitochondrial acetylation of SOD2↓, LCAD↓	**DH**: Sirtuin-3↓,AMPK ↓ **ET**:Sirtuin-3↑,AMPK↑	
8. Ko et al. [21]	**S:** Male Otuska Long– Evans Tokushima Fatty rats **A:** 28 weeks **DT:** Type-II	Climbing ladder	**Sp:** 20 Reps **F:** 5 days/week **P:** 12 weeks	**DH**: BW↑, glucose↑ **ET**: BW↓, glucose↓	**DH**: Membrane potential↓ **ET**: Membrane potential↑	**DH**: GLUT-4↓, PDHE1α↓ **ET**: GLUT-4↑, PDHE1α↑ **DH**: State-4 respiration↑, ROS↑ **ET**: State-4 respiration↓, ROS↓ **DH**: RCR ↓, ATP↓,SOD2↓ **ET**: RCR↑, ATP↑, SOD2↑ **DH**:UCP2↑ UCP3↑, **ET**: UCP2↓, UCP3↓	**DH**: PGC1α↓,TFAM↓ **ET**: PGC1α↑,TFAM↑	
9. Botta et al. [22]	**S:** Male C57BLKS/J mice with **A:** 6 weeks **DT:** Type-II	Motorized exercise wheel system	**Sp:** 5.2 m/min **F:** 5 days/week **P:** 3 weeks	**DH**: Blood glucose and BW changes **ET**: No changes observed after exercise		**DH**: TOM-70↓, VDAC-1 ↓ **ET**:TOM-70↑, VDAC-1↑ **DH**: ETC proteins lost complexes(I-V) **ET**: Complex I and activity II↓, IV ↑but III and V remain unchanged **DH**: SOD2↑ **ET**:SOD2↓	**DH**: mt DNA↓, PGC-1α↓ **ET**: mt DNA↑, PGC-1α↑ **DH**: TFAM↓, NRF 1 and 2↓ **ET**: No changes seen in the above transcription factors	**DH**: Signs of fission and fusion noted **ET**: No effect of exercise on fission and fusion
10. Oliveira et al. [29]	**S:** Male Wistar rats **A:** 6–8 weeks **DT:** Type-II	Motor-driven treadmill	**Sp:** 25 m/min **F:** 5 days/week **P:** 14 weeks	**DH**: Body weight↓, blood glucose↓ **ET**: Body weight↑, blood glucose↑	**DH**: Mitochondrial permeability ↑ **ET**: Mitochondrial permeability ↓ **DH**: Swelling↑ **ET**: Swelling↓	**DH**: State 3 and state 4 respiration↓ **ET**: State 3 and state 4 respiration↑ **DH**: Complex I and II activity ↓ **ET**: Complex I and II activity ↑ **DH**: RCR↓ **ET**: RCR↑	**DH**: TFAM↑ **ET**: TFAM↓	

**S**—sample of animal; **A**—age; **DT**—diabetes type, **Sp**—speed; **D**—duration of exercise; **F**—frequency of exercise; **P**—time period of exercise; **DH**—diabetic heart; **ET**—exercise training; **↑**—increase; **↓**—decrease. **Glycemic parameters and body weight:** (SG) serum glucose, (BW) body weight, hyperglycemia, and insulin resistance. **Mitochondrial physiological characteristics (MPC):** size, density, membrane potential, membrane permeability, cristae area, cristae size, and cross-sectional area. **(M0P): Mitochondrial oxidative phosphorylation:** (ETC) electron transport chain, (NADH) nicotinamide adenine dinucleotide, (SDH) succinyl dehydrogenase, (CCR) cytochrome c reductase, (CCO) cytochrome c oxidase, (4-HNE) 4 Hydroxynoneal, (NOX4) nitrogen oxide-4, p47 phox, p67 phox, (ROS) reactive oxygen species, (UCP) mitochondrial uncoupling proteins, (RCR) respiratory control ratio, (ATP) adenosine triphosphate, (CI, CII, CIII, CIV, and CV) ETC complexes, (IDH) isocitrate dehydrogenase, (OGDH) oxyglutarate dehydrogenase, (MCU) mitochondrial calcium uniporter, (OCR) oxygen consumption rate, oxidative stress, (FAO) fatty acid oxidation, acetylation, (LCAD) long-chain acyl-CoA dehydrogenase, (SOD2) superoxide dismutase, State 3 and 4 respiration, (GLUT-4) glucose transporter type-4, Tom-70, and (VDAC) voltage-dependent anion channel. **Mitochondrial biogenesis (MBG):** Irisin, (AMPK) AMP-activated protein kinase, (PGC-1α) peroxisome proliferator-activated receptor gamma co activator alpha, (NRF) nuclear respiratory factor, (16 RNAs) 16 ribonucleic acid, (mt DNA) mitochondrial DNA, ND1, ND6, (CYT-B) cytochrome B, (SSDBP1) single-stranded DNA binding proteins, Twinkle, Top1mt, (TFAM) mitochondrial transcription factor, Akt, (SSBP1) single-stranded binding proteins, and (SIRT3) Situin-3. **Mitochondrial dynamics (MD):** (Drp1) dynamin-related proteins, (Fis1) Fission 1, (MFF) mitochondrial fission factor, (MFP1) mitochondrial fission protein, fission, and fusion.

## References

[1] Ritchie R.H., Abel E.D. (2020). Basic mechanisms of diabetic heart disease. Circ. Res..

[2] Hafstad A.D., Boardman N., Aasum E. (2015). How exercise may amend metabolic disturbances in diabetic cardiomyopathy. Antioxid. Redox Signal..

[3] Huynh K., Bernardo B.C., McMullen J.R., Ritchie R.H. (2014). Diabetic cardiomyopathy: Mechanisms and new treatment strategies targeting antioxidant signaling pathways. Pharmacol. Ther..

[4] Sivitz W.I., Yorek M.A. (2010). Mitochondrial dysfunction in diabetes: From molecular mechanisms to functional significance and therapeutic opportunities. Antioxid. Redox Signal..

[5] Jin L., Geng L., Ying L., Shu L., Ye K., Yang R., Liu Y., Wang Y., Cai Y., Jiang X. (2022). FGF21-Sirtuin 3 Axis Confers the Protective Effects of Exercise Against Diabetic Cardiomyopathy by Governing Mitochondrial Integrity. Circulation.

[6] Fontana F., Limonta P. (2021). The multifaceted roles of mitochondria at the crossroads of cell life and death in cancer. Free Radic. Biol. Med..

[7] Dabkowski E.R., Baseler W.A., Williamson C.L., Razunguzwa T.T., Frisbee J.C., Hollander J.M., Pinti M.V., Fink G.K., Hathaway Q.A., Durr A.J. (2010). Mitochondrial dysfunction in the type 2 diabetic heart is associated with alterations in spatially distinct mitochondrial proteomes. Am. J. Physio. Heart Circul. Physiol..

[8] Kuznetsov A.V., Javadov S., Margreiter R., Grimm M., Hagenbuchner J., Ausserlechner M.J. (2019). The role of mitochondria in the mechanisms of cardiac ischemia-reperfusion injury. Antioxidants.

[9] Pizzino G., Irrera N., Cucinotta M., Pallio G., Mannino F., Arcoraci V., Squadrito F., Altavilla D., Bitto A. (2017). Oxidative Stress: Harms and Benefits for Human Health. Oxid. Med. Cell. Longev..

[10] Paulson D.J., Mathews R., Bowman J., Zhao J., Loganathan R., Bilgen M., Al-Hafez B., Zhero S.V., Alenezy M.D., Smirnova I.V. (1992). Metabolic effects of treadmill exercise training on the diabetic heart. J. Appl. Physiol..

[11] Fernandez-Marcos P.J., Auwerx J. (2011). Regulation of PGC-1α, a nodal regulator of mitochondrial biogenesis. Am. J. Clin. Nutr..

[12] Marin T.L., Gongol B., Zhang F., Martin M., Johnson D.A., Xiao H., Wang Y., Subramaniam S., Chien S., Shyy J.Y.-J. (2017). AMPK promotes mitochondrial biogenesis and function by phosphorylating the epigenetic factors DNMT1, RBBP7, and HAT1. Sci. Signal..

[13] Wright D.C., Geiger P.C., Han D.-H., Jones T.E., Holloszy J.O. (2007). Calcium induces increases in peroxisome proliferator-activated receptor γ coactivator-1α and mitochondrial biogenesis by a pathway leading to p38 mitogen-activated protein kinase activation. J. Biol. Chem..

[14] Din S., Konstandin M.H., Johnson B., Emathinger J., Völkers M., Toko H., Collins B., Ormachea L., Samse K., Kubli D.A. (2014). Metabolic dysfunction consistent with premature aging results from deletion of Pim kinases. Circ. Res..

[15] Zhang F., Lin J.J., Tian H.N., Wang J. (2024). Effect of exercise on improving myocardial mitochondrial function in decreasing diabetic cardiomyopathy. Exp. Physiol..

[16] Losón O.C., Song Z., Chen H., Chan D.C. (2013). Fis1, Mff, MiD49, and MiD51 mediate Drp1 recruitment in mitochondrial fission. Mol. Biol. Cell..

[17] Yu T., Robotham J.L., Yoon Y. (2006). Increased production of reactive oxygen species in hyperglycemic conditions requires dynamic change of mitochondrial morphology. Proc. Natl. Acad. Sci. USA.

[18] Yu T., Robotham J.L., Yoon Y. (2008). Mitochondrial fission mediates high glucose-induced cell death through elevated production of reactive oxygen species. Cardiovasc. Res..

[19] Makino A., Scott B., Dillmann W. (2010). Mitochondrial fragmentation and superoxide anion production in coronary endothelial cells from a mouse model of type 1 diabetes. Diabetologia.

[20] Trevellin E., Scorzeto M., Olivieri M., Granzotto M., Valerio A., Tedesco L., Fabris R., Serra R., Quarta M., Reggiani C. (2014). Exercise training induces mitochondrial biogenesis and glucose uptake in subcutaneous adipose tissue through eNOS-dependent mechanisms. Diabetes.

[21] Ko T.H., Marquez J.C., Kim H.K., Jeong S.H., Lee S., Youm J.B., Song I.S., Seo D.Y., Kim H.J., Won D.N. (2018). Resistance exercise improves cardiac function and mitochondrial efficiency in diabetic rat hearts. Pflügers Arch.-Eur. J. Physiol..

[22] Botta A., Laher I., Beam J., DeCoffe D., Brown K., Halder S., Devlin A., Gibson D.L., Ghosh S. (2013). Short term exercise induces PGC-1α, ameliorates inflammation and increases mitochondrial membrane proteins but fails to increase respiratory enzymes in aging diabetic hearts. PLoS ONE.

[23] Wang B., Zhao C., Wang Y., Tian X., Lin J., Zhu B., Zhou Y., Zhang X., Li N., Sun Y. (2023). Exercise ameliorating myocardial injury in type 2 diabetic rats by inhibiting excessive mitochondrial fission involving increased irisin expression and AMP-activated protein kinase phosphorylation. J. Diabetes.

[24] Wang S.Y., Zhu S., Wu J., Zhang M., Xu Y., Xu W., Cui J., Yu B., Cao W., Liu J. (2020). Exercise enhances cardiac function by improving mitochondrial dysfunction and maintaining energy homoeostasis in the development of diabetic cardiomyopathy. J. Mol. Med..

[25] Wang H., Bei Y., Lu Y., Sun W., Liu Q., Wang Y., Cao Y., Chen P., Xiao J., Kong X. (2015). Exercise Prevents Cardiac Injury and Improves Mitochondrial Biogenesis in Advanced Diabetic Cardiomyopathy with PGC-1α and Akt Activation. Cell. Physiol. Biochem..

[26] Bækkerud F.H., Salerno S., Ceriotti P., Morland C., Storm-Mathisen J., Bergersen L.H., Høydal M.A., Catalucci D., Stølen T.O. (2019). High Intensity Interval Training Ameliorates Mitochondrial Dysfunction in the Left Ventricle of Mice with Type 2 Diabetes. Cardiovasc. Toxicol..

[27] Veeranki S., Givvimani S., Kundu S., Metreveli N., Pushpakumar S., Tyagi S.C. (2016). Moderate intensity exercise prevents diabetic cardiomyopathy associated contractile dysfunction through restoration of mitochondrial function and connexin 43 levels in db/db mice. J. Mol. Cell. Cardiol..

[28] Mokhtar N., Lavoie J.P., Rousseau-Migneron S., Nadeau A. (1993). Physical training reverses defect in mitochondrial energy production in heart of chronically diabetic rats. Diabetes.

[29] Lumini-Oliveira J., Magalhães J., Pereira C.V., Moreira A.C., Oliveira P.J., Ascensão A. (2011). Endurance training reverts heart mitochondrial dysfunction, permeability transition and apoptotic signaling in long-term severe hyperglycemia. Mitochondrion.

[30] Chistiakov D.A., Shkurat T.P., Melnichenko A.A., Grechko A.V., Orekhov A.N. (2018). The role of mitochondrial dysfunction in cardiovascular disease: A brief review. Ann. Med..

[31] Parker A.M., Tate M., Prakoso D., Deo M., Willis A.M., Nash D.M., Donner D.G., Crawford S., Kiriazis H., Granata C. (2021). Characterisation of the Myocardial Mitochondria Structural and Functional Phenotype in a Murine Model of Diabetic Cardiomyopathy. Front. Physiol..

[32] Williamson C.L., Dabkowski E.R., Baseler W.A., Croston T.L., Alway S.E., Hollander J.M. (2010). Enhanced apoptotic propensity in diabetic cardiac mitochondria: Influence of subcellular spatial location. Am. J. Physiol. Heart C.

[33] Sloan R.C., Moukdar F., Frasier C.R., Patel H.D., Bostian P.A., Lust R.M., Brown D.A. (2012). Mitochondrial permeability transition in the diabetic heart: Contributions of thiol redox state and mitochondrial calcium to augmented reperfusion injury. J. Mol. Cell. Cardiol..

[34] Jubaidi F.F., Zainalabidin S., Mariappan V., Budin S.B. (2020). Mitochondrial Dysfunction in Diabetic Cardiomyopathy: The Possible Therapeutic Roles of Phenolic Acids. Int. J. Mol. Sci..

[35] Shen G.X. (2012). Mitochondrial dysfunction, oxidative stress and diabetic cardiovascular disorders. Cardiovasc. Hematol. Disord. Drug Targets.

[36] Gollmer J., Zirlik A., Bugger H. (2020). Mitochondrial mechanisms in diabetic cardiomyopathy. Diabetes Metabol. J..

[37] Bugger H., Abel E.D. (2010). Mitochondria in the diabetic heart. Cardiovasc. Res..

[38] Dludla P.V., Nkambule B.B., Tiano L., Louw J., Jastroch M., Mazibuko-Mbeje S.E. (2018). Uncoupling proteins as a therapeutic target to protect the diabetic heart. Pharmacol. Res..

[39] How O.-J., Aasum E., Severson D.L., Chan W.A., Essop M.F., Larsen T.S. (2006). Increased myocardial oxygen consumption reduces cardiac efficiency in diabetic mice. Diabetes.

[40] Viloria M.A.D., Li Q., Lu W., Nhu N.T., Liu Y., Cui Z.-Y., Cheng Y.-J., Lee S.-D. (2022). Effect of exercise training on cardiac mitochondrial respiration, biogenesis, dynamics, and mitophagy in ischemic heart disease. Front. Cardiovasc. Med..

[41] Lin C., Guo Y., Xia Y., Li C., Xu X., Qi T., Zhang F., Fan M., Hu G., Zhao H. (2021). FNDC5/Irisin attenuates diabetic cardiomyopathy in a type 2 diabetes mouse model by activation of integrin αV/β5-AKT signaling and reduction of oxidative/nitrosative stress. J. Mol. Cell Cardiol..

[42] Tao L., Huang X., Xu M., Yang L., Hua F. (2020). MiR-144 protects the heart from hyperglycemia-induced injury by regulating mitochondrial biogenesis and cardiomyocyte apoptosis. FASEB J..

[43] Tao L.-C., Wang T.-T., Zheng L., Hua F., Li J.-J. (2022). The role of mitochondrial biogenesis dysfunction in diabetic cardiomyopathy. Biomol. Ther..

[44] Xie Z., He C., Zou M.-H. (2011). AMP-activated protein kinase modulates cardiac autophagy in diabetic cardiomyopathy. Autophagy.

[45] Paramesha B., Anwar M.S., Meghwani H., Maulik S.K., Arava S.K., Banerjee S.K. (2021). Sirt1 and Sirt3 Activation Improved Cardiac Function of Diabetic Rats via Modulation of Mitochondrial Function. Antioxidants.

[46] Chen Y.H., Ta A.P., Chen Y., Lee H.C., Fan W., Chen P.L., Jordan M.C., Roos K.P., MacGregor G.R., Yang Q. (2023). Dual roles of myocardial mitochondrial AKT on diabetic cardiomyopathy and whole body metabolism. Cardiovasc. Diabetol..

[47] Karamanlidis G., Bautista-Hernandez V., Fynn-Thompson F., del Nido P., Tian R. (2011). Impaired mitochondrial biogenesis precedes heart failure in right ventricular hypertrophy in congenital heart disease. Circ. Heart Fail..

[48] García-Peña L.M., Abel E.D., Pereira R.O. (2024). Mitochondrial Dynamics, Diabetes, and Cardiovascular Disease. Diabetes.

[49] Hu L., Ding M., Tang D., Gao E., Li C., Wang K., Qi B., Qiu J., Zhao H., Chang P. (2019). Targeting mitochondrial dynamics by regulating Mfn2 for therapeutic intervention in diabetic cardiomyopathy. Theranostics.

[50] Zhou Y., Suo W., Zhang X., Liang J., Zhao W., Wang Y., Li H., Ni Q. (2023). Targeting mitochondrial quality control for diabetic cardiomyopathy: Therapeutic potential of hypoglycemic drugs. Biomed. Pharmacother..

[51] Trimarchi G., Pizzino F., Paradossi U., Gueli I.A., Palazzini M., Gentile P., Di Spigno F., Ammirati E., Garascia A., Tedeschi A. (2024). Charting the Unseen: How Non-Invasive Imaging Could Redefine Cardiovascular Prevention. J. Cardiovasc. Dev. Dis..

